# Mode of delivery and birth outcomes in relation to the duration of the passive second stage of labour: A retrospective cohort study of nulliparous women

**DOI:** 10.1371/journal.pone.0281183

**Published:** 2023-01-30

**Authors:** Maria Bjelke, Lars Thurn, Marie Oscarsson

**Affiliations:** 1 Department of Health and Caring Sciences, Linnaeus University, Kalmar, Sweden; 2 Department of Obstetrics and Gynaecology, Lund University, Lund, Sweden; Federal University of Sao Carlos: Universidade Federal de Sao Carlos, BRAZIL

## Abstract

**Objective:**

To investigate the mode of delivery and birth outcomes in relation to the duration of the passive second stage of labour in nulliparous women.

**Methods and findings:**

A retrospective cohort study of all nulliparous women (n = 1131) at two delivery units in Sweden. Maternal and obstetric data were obtained from electronic medical records during 2019. The passive second stage was defined as the complete dilation of the cervix until the start of the active second stage. The duration of the passive second stage was categorized into three groups: 0 to 119 min (0 to <2 h), 120–239 min (2- <4h) and ≥240 min (≥4h). Differences between the groups were examined using t-test and Chi2-tests and regression analyses were used to analyse adjusted odds ratio with 95% confidence intervals. The primary outcome was mode of delivery in relation to the duration of the passive second stage and the secondary outcomes covered a series of adverse maternal and neonatal birth outcomes. The rates of instrumental and caesarean deliveries increased as the duration of the passive second stage increased. A ≥4-hour duration of the passive second stage was associated with a nine-times increased risk of caesarean section, and a four-times risk of instrumental delivery compared to a duration of <2 hours in the adjusted analyses. No differences were found in the maternal birth outcomes. The risk of a 5-minute Apgar score <7 was increased in the 2-<4h group. A longer passive second stage was not associated with an increased risk of negative birth experience.

**Conclusions:**

Our study demonstrates an increased risk of operative delivery for a longer duration (>2h) of the passive second stage in nulliparous women, although most of the women gave birth by spontaneous vaginal delivery even after ≥4 hours. There was no evidence of an increased risk of adverse maternal outcomes in a longer duration of the passive second stage but there were indications of increased adverse neonatal outcomes. Assessment of fetal well-being is important when the duration of the passive phase is prolonged.

## Introduction

The second stage of labour is often defined as a single continuous phase, beginning at full dilation, and finishing at the birth. However, when the second stage is managed with delayed pushing it is divided into two phases: the passive second stage where the presenting part rotates and descends passively down the maternal pelvis, followed by the active second stage with pushing [1]. The contractions are longer and more frequent in the active second stage, causing uteroplacental circulation to reduce, unlike in the passive stage [2].

Prolonged second stage has been extensively studied, showing associations with an increased risk of operative delivery, post-partum haemorrhage (PPH), obstetric anal sphincter injury (OASI) and adverse neonatal outcomes [3–11]. There is no worldwide consensus regarding the definition of prolonged second stage and international organizations vary in their recommendations [12–14]. However, the two different phases in the second stage have not been separated in most studies and recommendations. A prolongation of the duration of each phase could have different consequences for the mother and the baby and should therefore be studied separately in greater detail. Studies with such a differentiation have primarily focused on the active second stage [15–18].

The World Health Organisation and the National Institute for Health and Care Excellence have in recent years suggested allowing longer duration for the second stage, if the condition of the woman and baby is satisfactory and there is documented progress of the labour [1, 13]. This use of an expectant management in the second stage has shown to decrease the rate of a primary caesarean delivery, but also to increase the rates of PPH, OASI and umbilical artery acidosis [10, 19]. There is insufficient knowledge about the consequences of a prolonged passive second stage due to the lack of separation between the passive and the active second stages. Clinical challenges may thus occur in recognising when to start a more active management and to determine the appropriate cut-off time for an instrumental delivery or caesarean section (CS). An accurate diagnosis of a prolonged passive second stage is important for evidence-based clinical decision-making and for the women who experience prolonged labour. An optimal management can allow the best opportunities for a spontaneous vaginal delivery (SVD) without an increased risk of adverse maternal and neonatal consequences.

The American College of Obstetricians and Gynaecologists has acknowledged that the second stage contains two phases, but does not provide any recommendations regarding the appropriate definition for a prolonged passive phase [20] and The International Federation of Gynecology and Obstetrics has identified a need for targeted research into the duration of the passive and active second stage and maternal and fetal outcomes [12]. A few previous studies have showed differences in birth outcomes regarding the duration of the passive second stage [21, 22] but this area needs to be further explored. The objective of this study was thus to investigate the mode of delivery and birth outcomes in relation to the duration of the passive second stage of labour in nulliparous women.

## Methods

**Fig 1:**
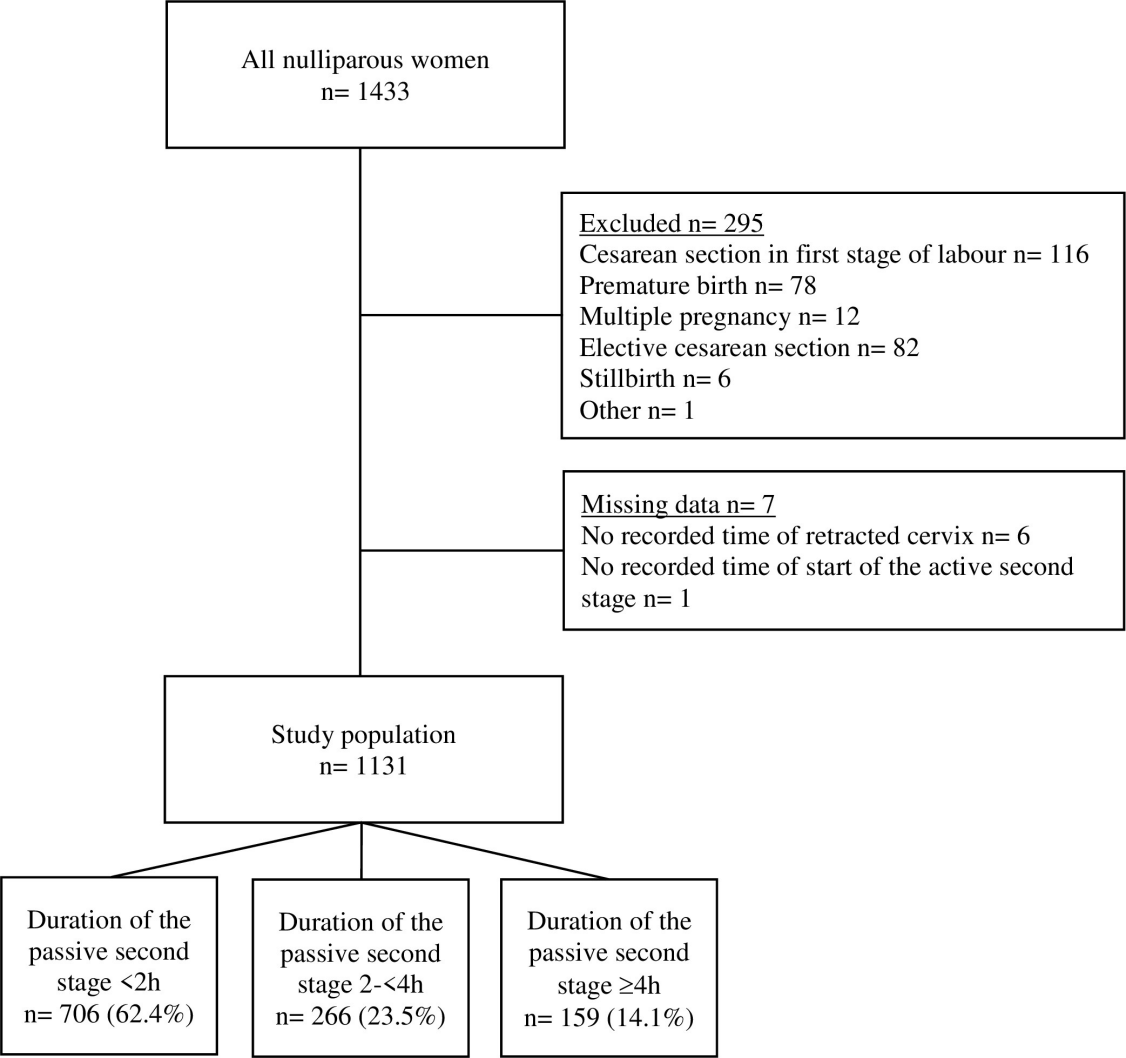
Flow chart of the study population.

A retrospective observational cohort design was chosen for this study, where the inclusion criteria were nulliparous women with singleton pregnancies in cephalic presentation at 37 gestational weeks or later who reached the second stage of labour. Stillbirths or missing data on retracted cervix or start of the active second stage were excluded (Fig 1). Detailed information on maternal, obstetric, and neonatal characteristics were obtained by a review of electronic medical records of all nulliparous women at two delivery units in Sweden between January 1 and December 31, 2019. The units had 2262 and 1527 births respectively during 2019, of which a total of 1433 women were nulliparous. There were no differences in maternal characteristics between the two hospitals. Healthcare during pregnancy and childbirth in Sweden is provided by the government without charge [23]. Midwives handle uncomplicated births independently and are supported by obstetricians if complications arise [24]. Obstetricians perform operative deliveries, including CS and instrumental deliveries.

### Definition of the passive second stage

The passive second stage was defined as the complete dilation of the cervix until the start of the active second stage, or complete dilation of the cervix until time of birth in case of an emergency caesarean before start of the active second stage. The general clinical practice in Sweden during the second stage is “delayed pushing”, instructing women to avoid pushing until there is an irresistible urge to push, or when the presenting part has descended to the perineum [25].

### Data collection and variable definition

The data were retrieved from medical charts and registered on a report form developed by the research group. The primary outcome was mode of delivery in relation to the duration of the passive second stage: spontaneous vaginal delivery, instrumental delivery or CS. The secondary outcomes were: PPH, OASI, clinical or laboratory verified intra- or postpartum infection, negative birth experience, low 5-minute Apgar score (<7) and admission to neonatal care unit (NICU). Furthermore, data on the following parameters were collected: maternal age and height, cohabiting, body mass index (BMI) in the first trimester, gestational age at delivery (calculated from the earliest obstetric ultrasound scan or the last menstrual period), occurrence of maternal diabetes (pre-existing or gestational), onset of active labour, length of second stage (passive- and active), use of epidural analgesia, use of oxytocin augmentation and birth weight.

The onset of active labour was defined as regular painful contractions, three to four in a ten-minute period, and a cervical dilation of three cm or more, in accordance with the Swedish standards in 2019 [26]. Cervical examinations were performed at least once every second hour but could also be more frequent depending on the progress of labour or maternal and fetal conditions. PPH was defined as an estimated blood loss of >1000ml within the first two hours after the birth. The blood loss was reported by the midwife or obstetrician by continuously measuring the amount of blood loss, either visually at low volumes of bleeding, or by weighing if abnormal bleeding occurred. Blood loss during CS was measured by the surgical suction device intraoperatively together with weighting cloths. The women received prophylactic oxytocin after the birth according to hospital guidelines (i.e. 8.3–16.6 μg oxytocin administered iv or im). All women in Sweden are asked to rate their overall birth experience using the Visual Analog Scale (VAS) score (usually 1–2 days after the birth). The reported VAS value is documented in the woman’s medical chart. The scale ranges from zero to ten, zero stands for the most negative experience possible and 10 for the most positive experience possible. A VAS score of three or less was defined as a negative birth experience. A low 5-minute Apgar score was defined as <7.

### Statistical analyses

The duration of the passive second stage was categorized into three groups: 0 to 119 min (0 to <2 h), 120–239 min (2- <4h) and ≥240 min (≥4h). Descriptive statistics for all study parameters were calculated for the three groups. Differences between the groups were examined using Chi2-tests or ANOVA. Regression analyses were executed to estimate the associations of the duration of the passive second stage (three groups), mode of delivery and birth outcomes. All maternal and neonatal outcomes, except mode of birth, were considered as dichotomous variables. Multivariable logistic regression models were used to control for confounders. The data was presented as crude (OR) and adjusted odds ratio (aOR) with 95% confidence interval (CI). Data analyses was conducted by IBM SPSS Statistics ® version 27. Ethical permission for the study was granted by the Regional Ethical Review Board in Linköping (Reg. No. 2017/447-31 and 2019/02844). Written informed consent was not required by the ethical committee. The data were retrieved from the medical charts and anonymized in the database. The STROBE guidelines for cohort studies were followed for this manuscript.

## Results

A total of 1131 nulliparous women were eligible for this cohort. The distribution between the three groups, 0 to <2 h, 2- <4h and ≥4h duration of the passive second stage is shown in Fig 1 and the characteristics of the study population are presented in Table 1 according to the three groups of the duration of the passive second stage. Women with longer passive second stages (2-<4h and ≥4h) tended to be older and had lower BMI. They were more likely to use epidural analgesia, oxytocin augmentation, give birth in a fetal malposition and their babies had higher birth weight. There was one shoulder dystocia and no maternal or neonatal deaths reported within the first two weeks after the birth in the study population. Six percent of the women (n = 70) gave birth in week ≥42+0.

**Table 1:** Maternal, obstetric and neonatal characteristics of the study population at baseline, presented as mean (SD) or n (%).

Characteristic	<2h	2-<4h	≥4h	p*	Post hoc Bonferroni^1^
	n = 706	n = 266	n = 159		
**Maternal**					
Age (y)	27.3±4.4	28.3±4.4	28.8±4.3	<0.001	B
BMI (kg/m2)	25.3±4.9	25.4±4.0	25.5±4.4	0.019	C
Height (cm)	166±6.4	165.4±6.4	166±6.4	0.388	-
Cohabitation during first trimester	627 (89)	250 (94)	147 (92.5)	0.028	B
Occurrence of diabetes ^2^	20 (2.9)	7 (2.6)	2 (1.9)	0.521	-
**Obstetric**					
Induction of labour	141 (20.0)	57 (21.4)	35 (22.0)	0.788	-
Use of epidural analgesia	380 (53.8)	207 (77.8)	141 (88.7)	<0.001	A
Oxytocin augmentation	304 (43.1)	215 (80.8)	154 (96.9)	<0.001	A
Duration of the active first stage (minutes)	305±228	380±226	384±225	<0.001	B
Duration of the active second stage (minutes) ^3^	41±27	46±30	35±28	<0.001	E
Prolonged rupture of membranes ^4^	113 (16.0)	72 (27.1)	45 (28.3)	<0.001	B
Fetal position at birth					
Occiput-anterior	678 (96.0)	249 (93.6)	140 (86.2)	<0.001	F
Other ^5^	28 (4.0)	17 (6.4)	22 (13.8)		
**Neonatal**					
Gestational (week)	39.6±1.2	39.8±1.2	39.7±1.4	0.030	D
Birthweight (gram), mean±SD	3427 ± 458	3556±435	3576±469	<0.001	B

Abbreviations: BMI, body mass index

Data are mean±SD or n (%).

* p-value of associations between duration of the passive second stage from Pearson’s chi-square test

^1^ Pairwise comparisons using the Pearson chi-square test or ANOVA (<0.05).

^2^ Pregestational or gestational

^3^ Vaginal deliveries

^4^ >18 hours

^4^ occiput-posterior, brow presentation, deep transverse, face presentation and other presentations

### Mode of delivery

**Fig 2:**
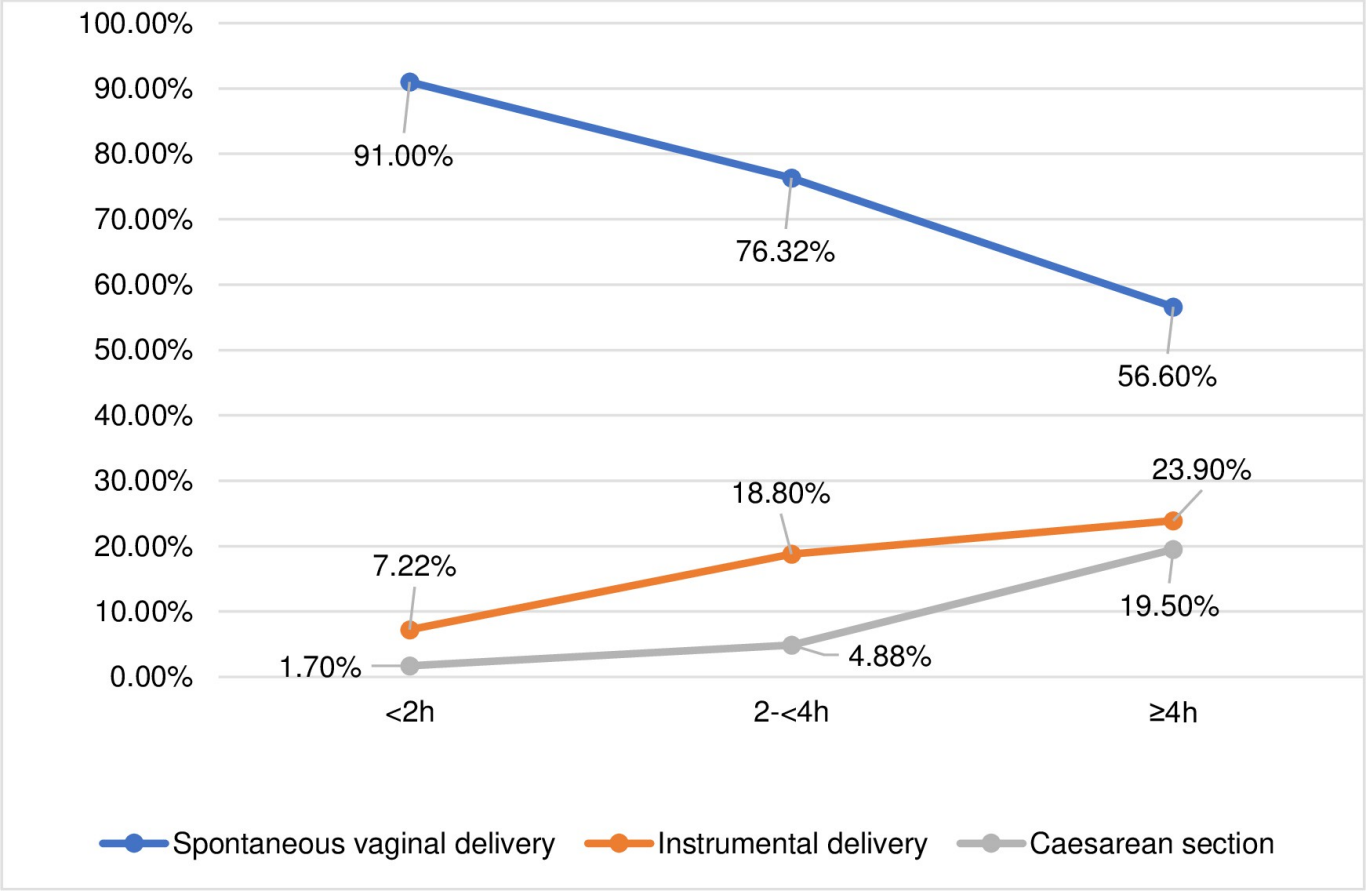
Mode of delivery in relation to the duration of the passive second stage.

The rates of SVD in the study population was 91% for women in the <2h group, 76% in the 2-<4h group and 57% for those who remained in the passive second stage for ≥4 hours. The rates of instrumental deliveries and CS increased as the duration of the passive second stage increased, seen in Fig 2. These results persisted in the multivariable logistic regression analyses, seen in Table 2.

**Table 2:** Mode of delivery in relation to the duration of the passive second stage: Crude odds ratio and multivariable adjusted logistic regression models with 95% CI.

	Duration of the passive second stage		
**Mode of delivery**	**<2h**	**2-<4h**	**≥4h**
	**n = 706**	**n = 266**	**n = 159**
Spontaneous vaginal delivery	n = 643	n = 203	n = 90
OR	1	0.32 (0.22–4.6) *	0.13 (0.09–0.19) *
aOR^1^	1	0.49 (0.31–0.78) *	0.27 (0.16–0.47) *
Instrumental delivery	n = 51	n = 50	n = 38
OR	1	2.97 (1.96–4.52) *	4.03 (2.54–6.41) *
aOR^1^	1	1.99 (1.24–3.2) *	3.42 (1.99–5.87) *
Caesarean section	n = 12	n = 13	n = 31
OR	1	2.97 (1.34–6.60) *	14.01 (7.01–28.00) *
aOR^1^	1	1.78 (0.76–5.16)	8.86 (4.21–18.67) *

Abbreviations: OR, odds ratio; aOR, adjusted odds ratio

*p<0.05

^1^ Adjusted for maternal age, height, smoking, BMI, cohabitation, birthweight, gestational age, induction of labour, use of epidural analgesia, oxytocin augmentation, active second stage >60 min

### Birth outcomes

Tables 3 and 4 present the associations between the duration of the passive second stage and adverse maternal birth outcomes. Associations were found between 2-<4h and ≥4 h duration of the passive second stage and all adverse outcomes except OASI in the unadjusted analyses but no associations were found after adjusting for confounders.

**Table 3:** Birth outcomes the study population in relation to the passive second stage presented as n (%).

Birth outcome	<2h	2-<4h	≥4h
	n = 706	n = 266	n = 159
PPH ^1^	56 (7.9)	33 (12.4)	22 (13.8)
OASI ^2^	33 (4.7)	13 (5.1)	7 (4.4)
Clinical or laboratory verified intra- or postpartum infection ^3^	30 (4.2)	24 (9.0)	13 (8.2)
Negative birth experience ^4^^,^ ^5^	53 (7.5)	32 (12.0)	25 (15.7)
5-min Apgar score <7 ^6^	9 (1.3)	9 (3.4)	4 (2.5)
Admission to NICU	49 (6.9)	26 (9.8)	19 (11.9)

Abbreviations: PPH, post-partum haemorrhage: OASI, obstetric anal sphincter injury: NICU, neonatal intensive care unit.

* p-value of associations between duration of the passive second stage from Pearsons chi-square test

^1^ >1000ml

^2^ Vaginal deliveries

^3^ Within two weeks after the delivery

^4^ Visual analogue scale ≤3

^5^ Data from 982 births

^6^ Data from 1126 births

**Table 4:** Maternal and neonatal outcomes in relation to the duration of the passive second stage: Crude odds ratio and multivariable adjusted logistic regression models with 95% CI.

	Duration of the passive second stage		
**Birth outcome**	**<2h**	**2-<4h**	**≥4h**
	**n = 706**	**n = 266**	**n = 159**
PPH			
OR	1	1.66 (1.05–2.61) *	1.86 (1.10–3.15) *
aOR^1^	1	1.45 (0.88–2.47)	1.23 (0.60–2.51)
OASI ^2^			
OR	1	1.05 (0.54–2.02)	0.94 (0.41–2.16)
aOR ^1^	1	0.67 (0.32–1.40)	0.64 (0.25–1.64)
Intra or postpartum infection			
OR	1	2.24 (1.28–3.90) *	2.01 (1.02–3.94) *
aOR ^1^	1	2.17 (1.10–4.26) *	1.58 (0.63–4.00)
Negative birth experience			
OR	1	1.63 (1.02–2.62) *	2.38 (1.42–4.00) *
aOR ^1^	1	0.85 (0.48–1.52)	0.57 (0.26–1.26)
5-min Apgar <7 ^3^			
OR	1	2.71 (1.06–6.90) *	1.99 (0.60–6.54)
aOR ^1^	1	4.24 (1.32–13.61) *	1.62 (0.26–9.92)
Admission to NICU			
OR	1	1.45 (0.88–2.39)	1.82 (1.04–3.19) *
aOR ^1^	1	1.71 (1.01–3.08) *	2.30 (1.12–4.72) *

Abbreviations: PPH, post-partum haemorrhage; OASI, obstetric anal sphincter injury; OR, odds ratio; aOR, adjusted odds ratio

*p<0.05

^1^ Adjusted for maternal age, height, smoking, BMI, cohabitation, birthweight, gestational age, induction of labour, use of epidural analgesia, oxytocin augmentation, active second stage >60 min, operative delivery

^2^ Vaginal deliveries

^3^ Data available from 1126 labours

Table 4 presents the crude and adjusted association between the duration of the passive second stage and the neonatal outcomes. The risk of a low 5-minute Apgar score had a fourfold increase in the 2-<4h duration-group (aOR 4.24, 95% CI 1.32–13.61) and the risk of admission to NICU increased in the 2-<4h (1.71, 95% CI 1.01–3.08) and in the ≥4 h group (2.30, 95% CI 1.12–4.72). The incidence of adverse neonatal outcomes in the study population was a rate of 8.3% (n = 94) admittance to NICU and 1.9% (n = 22) low 5-minute Apgar score. The most common reason for admittance to NICU was respiratory distress (n = 25) and clinical or laboratory verified neonatal infection (n = 23).

### Birth outcomes in relation to the mode of delivery

**Fig 3:**
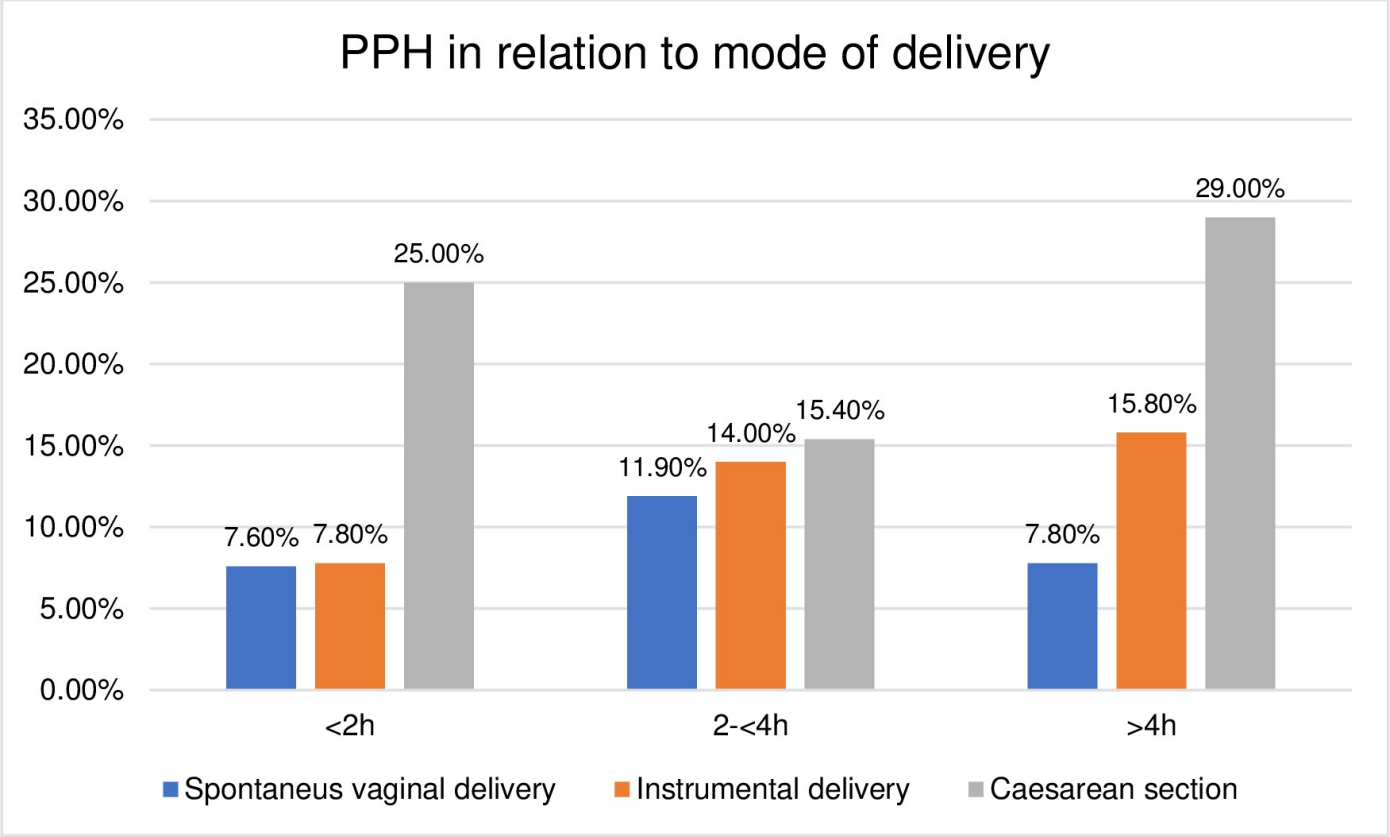
Histogram of PPH in relation to mode of delivery.

**Fig 4:**
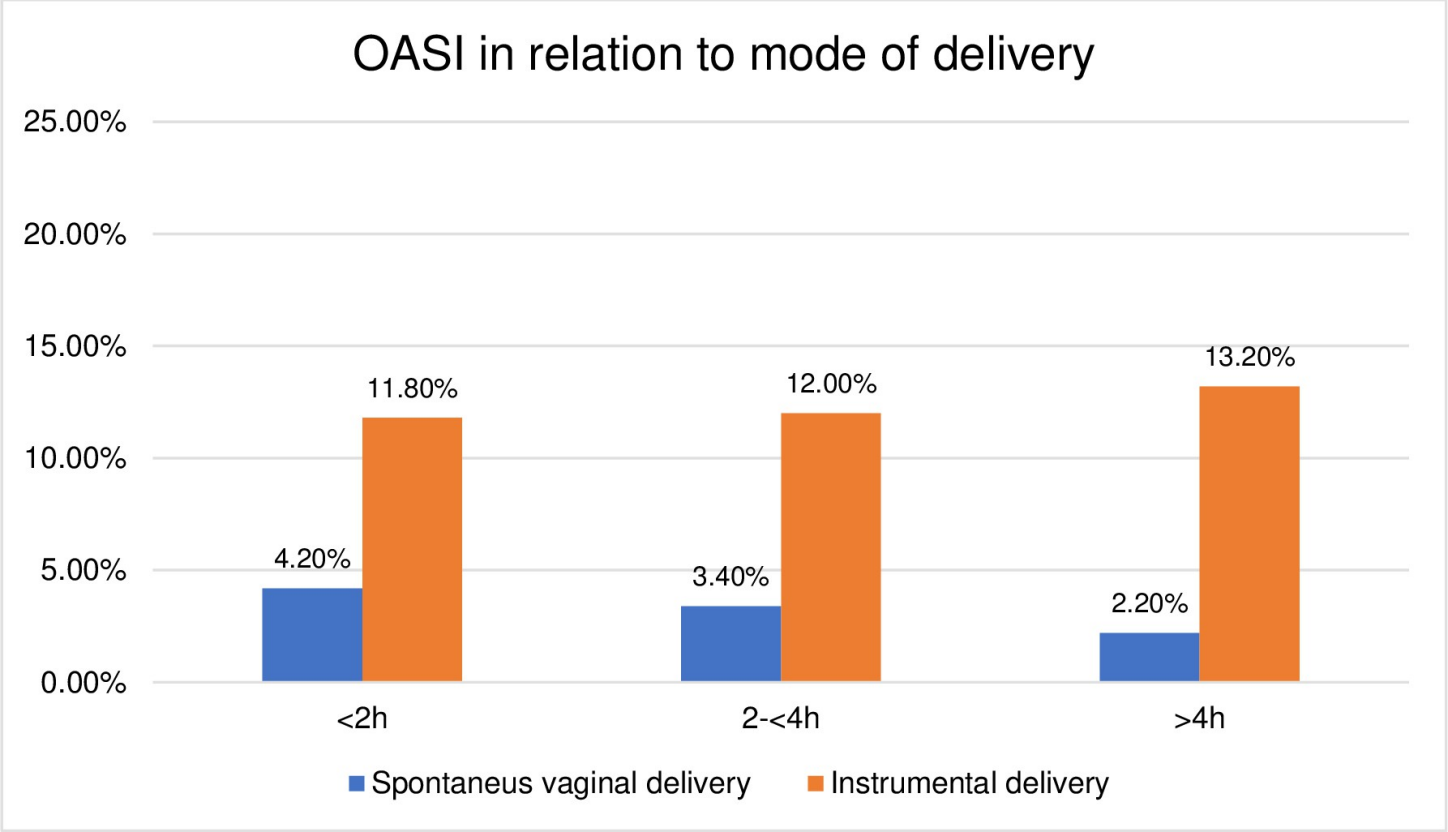
Histogram of OASI in relation to mode of delivery (vaginal deliveries).

**Fig 5:**
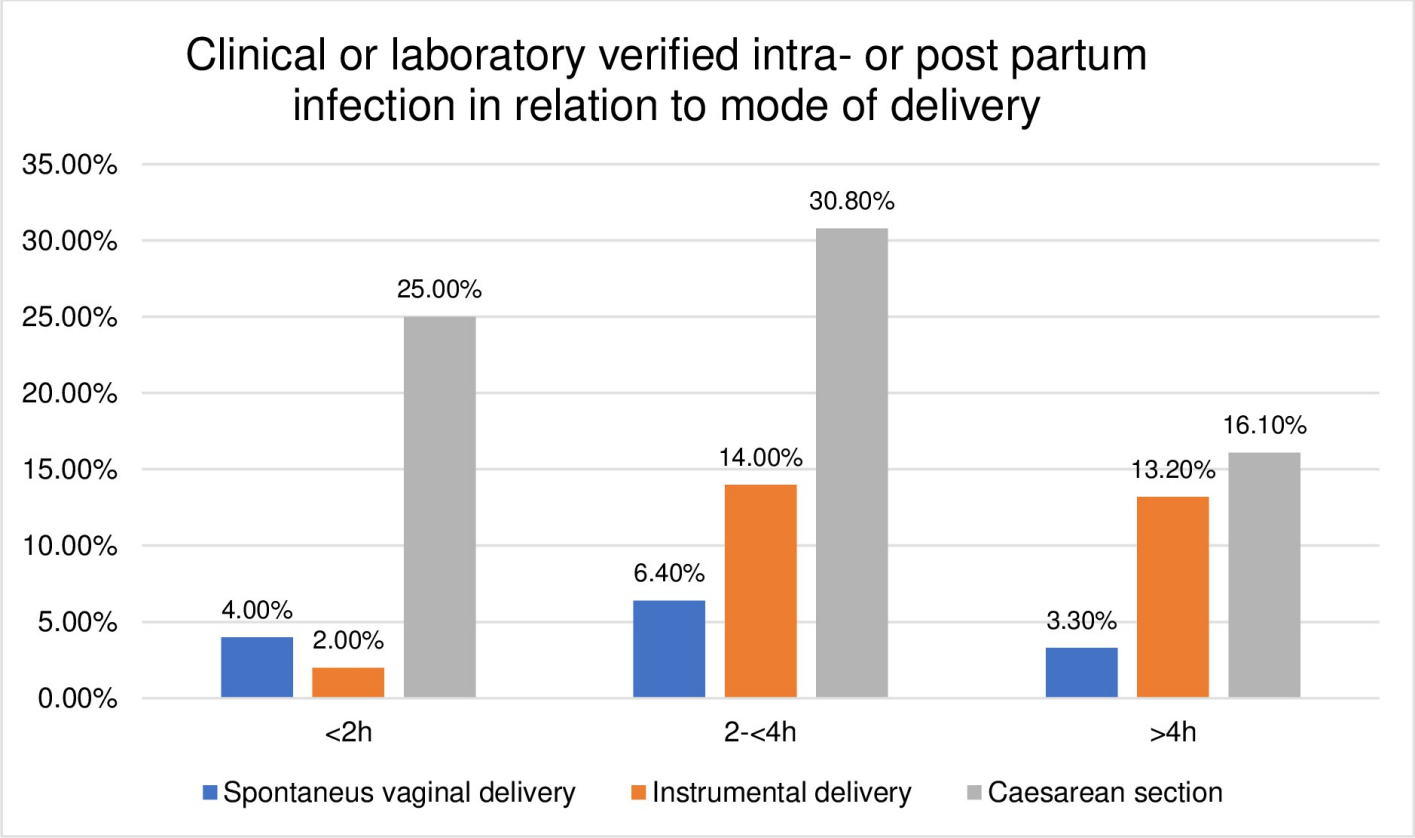
Histogram of intra or post-partum infection in relation to mode of delivery.

**Fig 6:**
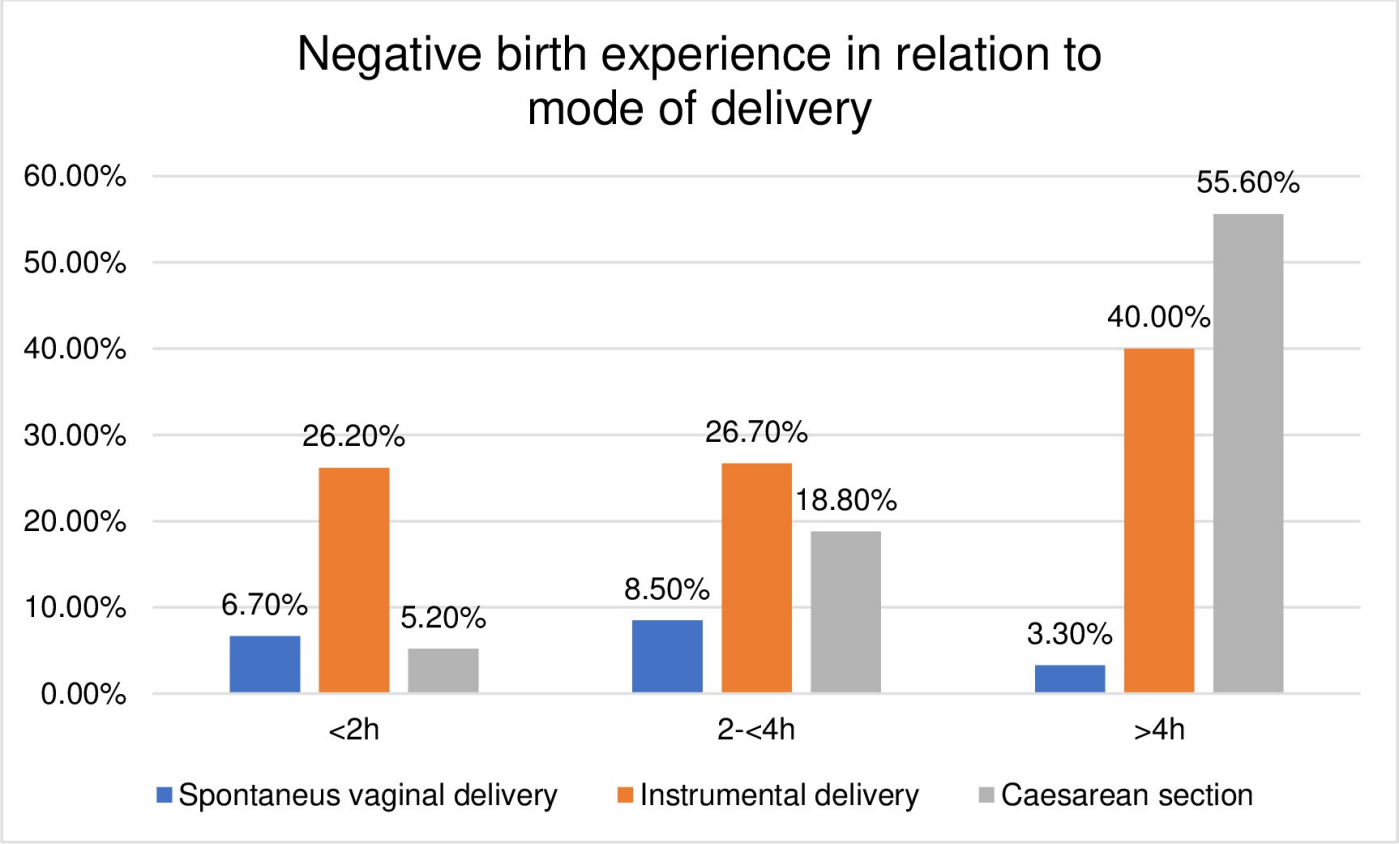
Histogram of negative birth experience in relation to mode of delivery.

**Fig 7:**
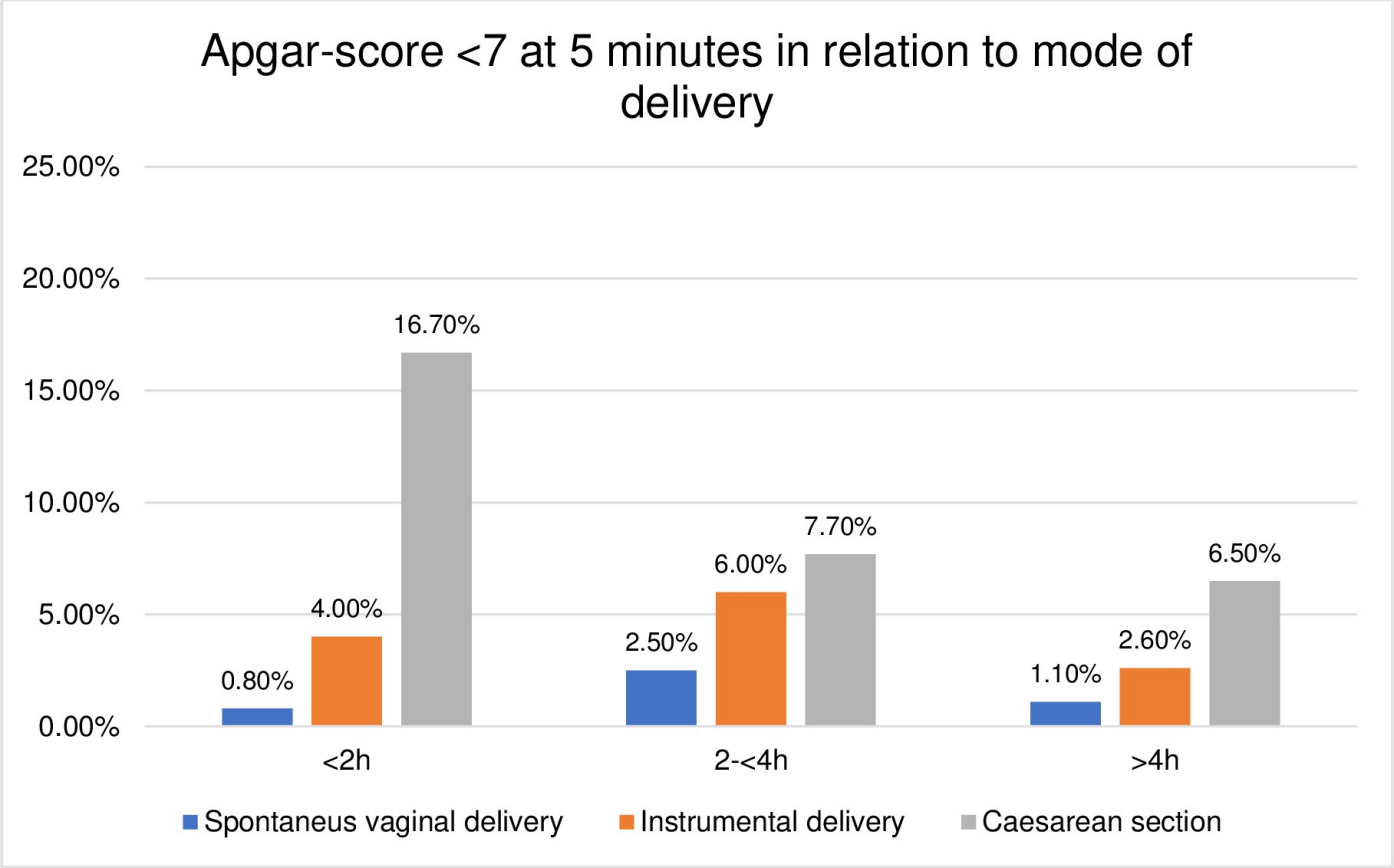
Histogram of Apgar score <7 at 5 minutes in relation to mode of delivery.

**Fig 8:**
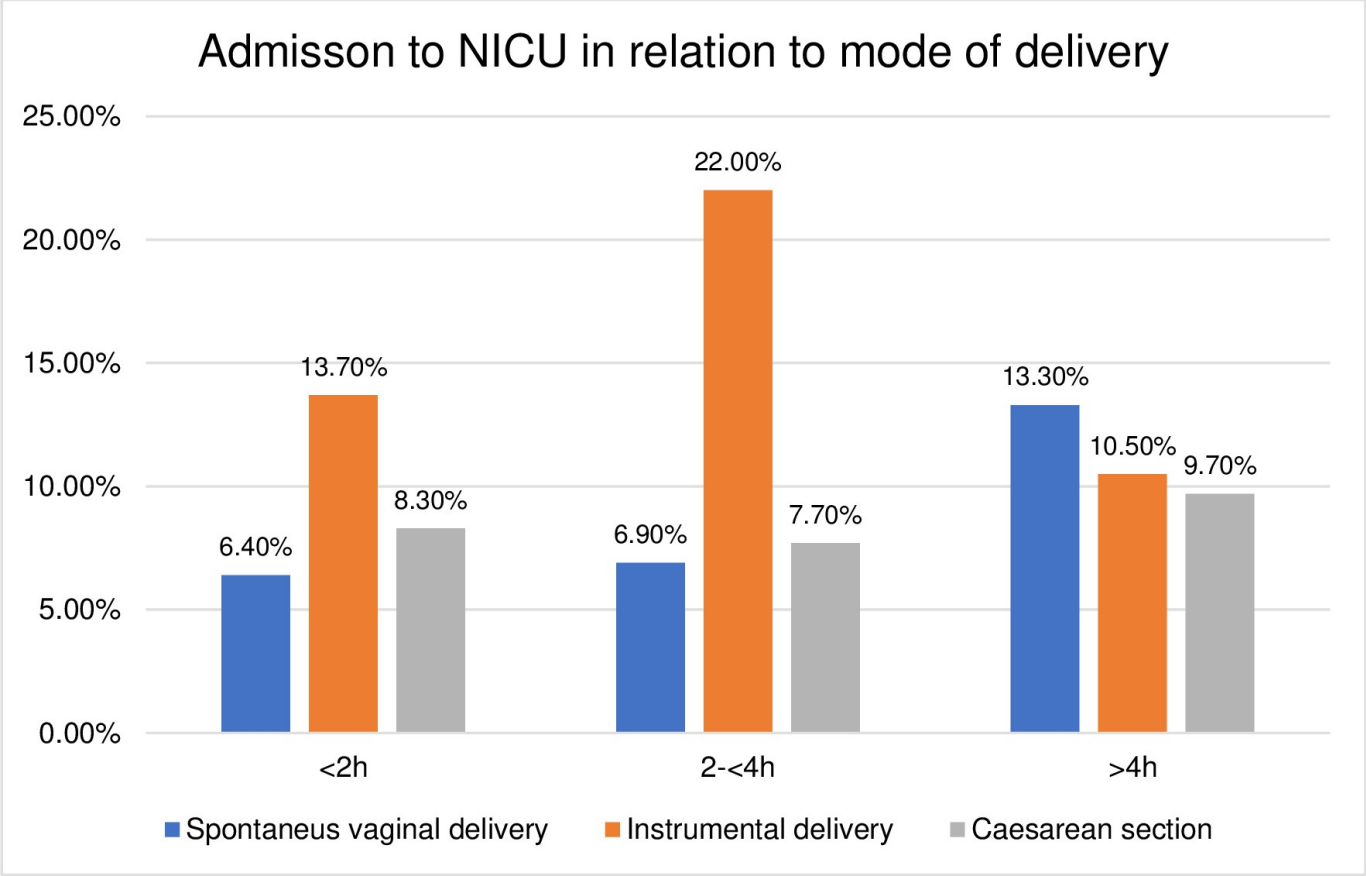
Admission to NICU in relation to mode of delivery.

The duration of the passive second stage and birth outcomes were further explored according to their relation to the mode of delivery. The distribution was found to be skewed towards higher rates of adverse birth outcomes in operative deliveries compared to SVD, shown in Figs 3–8. The women who experienced a passive second stage of ≥4 h, and birth by SVD had a PPH rate of 7.8% in comparison to 29% of the women delivered by CS (p = 0.009), Fig 3.

## Discussion

We found that a longer duration of the passive second stage was associated with an increased risk of operative delivery in this retrospective cohort-study, although most of the women gave birth by spontaneous vaginal delivery even after ≥4 hours. No differences were found in the maternal birth outcomes. The risk of a 5-minute Apgar score <7 was increased in the 2-<4h group and the risk of admission to NICU was increased in the 2-<4h and ≥4-hour group.

This study is to our knowledge the first to report on mode of delivery in relation to the duration of the passive second stage. Previous studies have described the second stage as one continuous phase without a distinction between passive descent and active pushing or focusing only on the active phase of the second stage [4, 5, 7, 9, 18, 27]. Instrumental deliveries are known to increase the risk of OASI [28, 29], which was also shown in this study. A second stage CS has higher incidences of complications compared to vaginal deliveries [30, 31] and a first stage CS due to the deeply engaged fetal head [32–34]. Allowing an extension of the duration of the second stage has previously shown to decrease the rate of CS but also to increase the rate of PPH and OASI [10, 19], but these studies lack differentiation between the two different phases of the second stage and the results may be affected by the duration of the active second stage. Our study did not show any associations between longer duration of the passive second stage, OASI and PPH. Similar results have been shown in a French study regarding the risk of PPH [21]. In a comparison of PPH after giving birth by SVD or CS among the women with the longest passive second stages in our study, ≥4 h, there was a greater frequency of PPH for the CS births, 29%, while only 7.8% for SVD (p = 0.009). We suggest that these observations should be included in the decision-making process for operative deliveries due to longer duration of the passive second stage.

Previous research has shown conflicting results regarding the prolonged second stage and neonatal outcomes [7, 35, 36]. A Swedish study has reported a negative effect on neonatal outcomes when the passive second stage was combined with an active second stage ≥ 45 min [22]. We showed increased risks affecting low 5-minute Apgar score (2-<4h group) and admission to NICU (2-<4h and ≥4-hour group). The passive second stage does not contain the same physiological processes as the active but does for unclear reasons generate an increased risk of adverse neonatal outcomes when it is prolonged, although adverse outcomes were still rare in the study population. The observational study design made it not possible to conclude other causal associations that might be possible with a larger study population. Almost all (97%) of the women in the ≥4 h group received oxytocin augmentation, associated with negative effects related to the risk of hyperstimulation. Clinical assessment of fetal well-being is essential when the duration of both phases in the second stage is prolonged, especially when oxytocin augmentation is prevalent, and the benefits of increased chance of SVD should be weighed against potential increases in neonatal risks.

This study was conducted in Sweden where delayed pushing is the standard management for the second stage of labour. Globally, both immediate and delayed pushing are commonly used, and the optimal management for the second stage is unclear [25]. An American multicenter randomized clinical trial investigated the difference between the two approaches among nulliparous women receiving epidural analgesia. The definition for delayed pushing was instructing the women to wait 60 minutes before pushing. Delayed pushing reduced the time of active pushing at the expense of a longer total second stage, showed a higher rate of chorioamnionitis and neonatal acidemia but no difference in mode of delivery. The authors conclude that immediate pushing for women with epidurals may be preferred due to the increased maternal and neonatal complications of delayed pushing [37]. Our study involves women with and without epidural analgesia, also showing increased risks of neonatal complications although there is a difference in the definition of delayed pushing. Swedish guidelines on labour management do not account for the presence of epidural analgesia unlike some international guidelines and immediate pushing has to our knowledge not been studied in this obstetrical context.

Mode of delivery is a significant indicator for women’s birth experience [38, 39]. SVD is associated with the highest-rated birth experience among nulliparous women [40, 41]. A longer passive second stage was not associated with an increased risk of negative birth experience in the multivariable regression analyses in this study, but women with operative deliveries reported a higher frequency of negative birth experience compared to women with SVDs, which has been previously reported in an American study [42]. The VAS scale has limitations due to the simplified measure of the complex nature of a birth experience, but on the other hand, is an established tool for measuring birth experience in clinical practices in Sweden [43, 44]. Other validated tools such as W-DEQ [45] or CEQ2 [46] might have provided a more thorough evaluation of the women’s birth experience. Another limitation is that the birth experience was evaluated within the first days after the birth, the rating of birth experience has shown to change over time [47]. Women’s experiences during the passive second stage should also be further explored using a qualitative design is required to better understand what initiates positive or negative experience and what can facilitate improvements of labour care.

All women should be offered labour care where factors associated with shorter labour duration and increased possibility of SVD is included. This is especially important when risk factors for prolonged labour is prevalent. Epidural analgesia and fetal malposition has previously been associated with an increased risk of prolonged passive second stage (≥2 hours) [48] and persistent occiput posterior position is associated with increased risk of operative delivery [49]. These factors need to be addressed in the clinical evaluation of longer duration of the passive second stage. Women who receive a continuity model of care during their pregnancy and women receiving continuous support during labour and birth are less likely to have epidural analgesia and instrumental delivery, and their chances of a SVD is increased [50, 51]. A Swedish study has shown that immersion in water is associated with shorter duration of the passive second stage (nulli- and multiparous women) [52], possibly due to the absence of epidurals in waterbirths.

### Strengths and limitations

A major strength of this study is the low level of missing data and the annual cohort of all nulliparous women at two hospitals. The manual chart review provided detailed information and strengthened the validation compared to register data by decreasing the risk of missing information due to lack of documentation of diagnostic codes or misclassifications. Adjustments for multiple important confounders were performed during the statistical analyses.

The retrospective design raises the possibility of biases inherent to this type of study. The cohort did not contain information on presence of thrombosis prophylaxis, selective serotonin reuptake inhibitors, blood transfusions, or polyhydramnios. The transition between the first stage and the passive second stage cannot be precisely established. Labour data is complex and recorded data depended on the timing of cervical examination [13]. We cannot rule out the possibility that some durations of the passive second stage are understated and were in fact longer.

There is no universal consensus regarding the definition of a prolonged passive second stage. A definition for prolongation may be used exclusively when instrumental delivery or CS is indicated [14] while others use it when oxytocin augmentation is initiated [53]. This split view of the definition may contribute to the current conflicting evidence regarding the prolonged second stage, together with the issue of not separating the passive and the active second stage, which is necessary when delayed pushing is the standard management for the second stage. We addressed the issue of a cut-off time for an operative delivery due to a prolonged passive second stage in the light of maternal and neonatal safety in this study. A previous study from our research group has shown a rate of 38% for the duration of the passive second stage ≥2h [48]. We thus chose to categorize the population into three groups (0 to <2 h, 2- <4h and ≥4h) in this study to increase the clinical relevance for decision-making on expectant management or operative delivery. The optimal cut-off time for operative delivery due to a prolonged passive second stage remains uncertain and an individual approach to second stage management to include the complex process of birth may instead be required in the future.

## Conclusion

Our data provide new information on the importance of dividing the second stage into a passive and an active phase. A longer duration (>2h) of this phase was associated with an increased risk of operative delivery, although there was no evidence of increased risk of adverse maternal outcomes in a longer duration of the passive second stage. The decision to perform an instrumental delivery or CS should therefore be weighed against the option of continuing labour when maternal and fetal status is reassured. Attention should also be paid to a preventive labour management with interventions that are associated with a greater chance of SVD.

## Supporting information

S1 Checklist STROBE statement—checklist of items that should be included in reports of *cohort studies*. (PDF)

## Abbreviations

aOR: adjusted odds ratio
BMI: body mass index
CI: confidence interval
CS: caesarean section
NICU: neonatal intensive care unit
OASI: obstetric anal sphincter injury
OR: odds ratio
PPH: postpartum haemorrhage
SVD: spontaneous vaginal delivery
